# Testosterone Therapy in Men with Advanced Prostate Cancer: Too Many Unknowns for Safe Use

**DOI:** 10.1089/andro.2021.0015

**Published:** 2021-07-21

**Authors:** Jesse Ory, Ranjith Ramasamy

**Affiliations:** Department of Urology, University of Miami, Miami Florida, USA

Accepted practice surrounding the use of exogenous testosterone in the presence of treated or untreated localized prostate cancer has changed dramatically over the past decade. Recent evidence has failed to show an increased risk of *de novo* disease in the general population,^1^ or of progression or recurrence in men after definitive therapy or on active surveillance for localized disease.^2^ This has culminated in recent American Urological Association guidelines on testosterone therapy (TT) reinforcing the lack of evidence connecting TT to *de novo* prostate cancer, and supporting the safety of TT in men with treated prostate cancer—primarily in men with low or intermediate risk disease.^1^

This is all predicated on the saturation hypothesis: that prostate cells will not respond to testosterone above a certain “saturation” threshold, thought to be between 100 and 200 ng/dL.^3^ In this article, TT in men with biochemical recurrence and metastatic prostate cancer: initial observations by Morgentaler *et al.*,^4^ the authors recognize the lack of evidence in men with advanced disease, the morbidity caused by androgen deprivation, and hypothesize that based on prior evidence in localized disease, that testosterone in these men may be safe.

This hypothesis is predicated on the assumption that the saturation hypothesis applies to men with mutated prostate cancer cell lines, as often happens with metastasis or advanced disease.^5,6^ This is likely not the case, as one can see from data in this series. Prostate-specific antigen increased by >10 points in men with biochemical recurrence and metastatic disease, in men with a mean testosterone of 204ng/dL (range 3–629), above the proposed saturation threshold. In addition, 5 of 22 men died, with 3 dying while on testosterone. Only 10 men of 22 had follow-up imaging, 3 of which showed progression. This seems in line with existing evidence in advanced prostate cancer: increasing testosterone >20 ng/dL in men with metastatic or recurrent prostate cancer is associated with worse outcomes.^7^ Regardless, the authors have made it clear that men were thoroughly counseled as to the potential risks of TT, and all had spoken to their oncologists beforehand.

Although there is no doubt that these men likely had symptoms severely impacting their quality of life, these data stress the importance of involving the patient’s oncologist and thoroughly counseling them as to the risks of pursuing testosterone in this disease state. Some men had normal serum testosterone in this study, highlighting the importance of searching for other causes of their symptoms before starting them on a medication that may hasten their death. Research into the use of human chorionic gonadotropin or selective estrogen receptor modulators in this population may be worthwhile, as the mutated androgen receptor would be exposed to less testosterone than with exogenous T. Ultimately, until more data are collected, patients must balance the prospect of symptom improvement with the real possibility of progression or death. A multidisciplinary approach should be pursued in these difficult cases, and more *in vitro* research should be conducted before further use in patients.

## Abbreviation Used

TT: testosterone therapy
